# Terri Grodzicker: the quintessential scientist–Editor

**DOI:** 10.1101/gad.350442.123

**Published:** 2023-01-01

**Authors:** Danny Reinberg

**Affiliations:** Howard Hughes Medical Institute, Smilow Research Center, Department of Biochemistry and Molecular Pharmacology, New York University Langone School of Medicine, New York, New York 10016, USA

As Terri Grodzicker transitions from her decades-long position as Editor of *Genes & Development*, I salute her service to the scientific community, her integrity, and her sense of fairness that marked her career. I also proudly acknowledge our friendship that has grown steadily throughout our scientific exchanges.

Under Terri's direction, *G&D* became a very important journal; acceptance within its covers spoke of the topical nature of the study as well as its well-conceived and well-performed experiments; and such acceptance was quite desirable. *G&D* publications were of high quality, reflecting Terri's goals of quality in lieu of quantity. In fact, the success of *G&D* may have encouraged Ben Lewin to initiate the journal *Molecular Cell*. Interestingly, when Ben Lewin announced the inception of *Molecular Cell* as a new journal of “Cell Press,” participants at the Cold Spring Harbor Laboratory Transcription meeting were sounding the death knell for *G&D*. But, au contraire, Terri's commitment and rigorously high standards kept *G&D* at the cutting edge of the different disciplines of gene expression and development and at the forefront of scientific journals.

Terri strongly impacted the scientific content of *G&D*. She stayed on top of the new directions being pursued by molecular biologists and the impetus of such new directions—whether well founded or not—by attending many conferences. I interacted with her often at these meetings and was privy to Terri's valuable comments and professional nature. I developed the utmost respect for her opinions. Terri was generally open to advice and would sometimes reconsider her stance on an issue. Importantly, Terri was very open to considering review articles on topics that were underappreciated or underrepresented yet conceivably of importance to the field. She enthusiastically invited scientists to write reviews and she read them, often inserting her own perspective and advice in the responding cover letter to have the review be as excellent as possible. She would also reject a review, albeit invited, if it did not match her standards!

As the Editor of *G&D*, Terri was very effective and maintained a tough but fair stance. Terri did not mince words; she was eminently clear, leaving nothing to one's hopes or imagination, as expected from someone that grew up in Brooklyn, NY. She showed no favoritism, yet she was willing to accommodate some issues. So, for example, if a manuscript from my lab had gone out to be reviewed by another journal and the reviews, while good, took so long to receive that the original journal had lost interest in publishing our study, Terri would, in some cases, expedite review of the study in *G&D*. In fact, when asked whether the reviewer comments from the earlier journal could be sent to her, she would not even look at the original reviews, stating, “How do I know they weren't written by your grandmother?”! When placed in the quandary of very disparate reviews of a paper, she would convey the gist of the other reviews to the opposing reviewer, giving the opportunity to reconsider; this was incredibly fair to both the authors of the manuscript and the reviewers themselves. Indeed, Terri stood for balance and accuracy in science.

While Terri is tough, she is also professional, and a very sensitive human being. Through our association over the decades, Terri and I have become close friends. She extended her ear too many times to mention with great interest and supportive advice for any of my scientific or personal issues. She manages to get a chuckle or laugh out of me time and again, as Terri has a marvelous quick wit and a Brooklyn-born sense of humor that defines her in the most wonderful way. I know whatever path she chooses to engage in now, she will do so with gusto and integrity, and will be on the up-and-up all the way!

